# Malnutrition Defined by Geriatric Nutritional Risk Index Predicts Outcomes in Severe Stroke Patients: A Propensity Score-Matched Analysis

**DOI:** 10.3390/nu14224786

**Published:** 2022-11-12

**Authors:** Ying Chen, Xinguang Yang, Yingying Zhu, Xiaoni Zhang, Jingxian Ni, Yi Li

**Affiliations:** 1 Department of Neurology, Sun Yat-sen Memorial Hospital, Sun Yat-sen University, Guangzhou 510000, China; 2 Division of Clinical Research Design, Sun Yat-sen Memorial Hospital, Sun Yat-sen University, Guangzhou 510000, China

**Keywords:** severe stroke, geriatric nutritional risk index, malnutrition, mortality, intensive care unit, propensity score matching method

## Abstract

Background: Malnutrition’s prognostic impact in patients with severe stroke requiring ICU admission is not well known. This study aimed to assess the nutritional status of severe stroke patients using the geriatric nutritional risk index (GNRI) and examine the association of GNRI with mortality in that population. Methods: We identified 1145 severe stroke patients requiring ICU admission from the Medical Information Mart for Intensive Care (MIMIC-III) database and divided them into low GNRI (≤98) or high GNRI (>98) groups. We used a propensity score matching (PSM) method to reduce confounding. Cox proportional hazards regression and restricted cubic splines were used to elucidate the association between GNRI and mortality. Hazard ratios (HR) and 95% confidence intervals (CI) were calculated. Results: A total of 397 (35%) patients were in the low GNRI group (at risk of malnutrition). After PSM, patients in the low GNRI group still suffered higher mortality compared with the high GNRI group at 28 days (27.9 vs. 20.8%), 90 days (35.5 vs. 25.7%), and 1 year (43.4 vs. 30.9%) (*p* < 0.05). A low GNRI was significantly associated with an increased mortality (HR: 1.38, 95% CI 1.03–1.86 in 28 days; HR: 1.45, 95% CI 1.11–1.89 in 90 days; HR: 1.51, 95% CI 1.19–1.92 in 1 year). Sensitivity analyses yielded consistent results. Restricted cubic splines showed a progressively decreasing risk of mortality with increasing GNRI scores up to 110, approximately. Conclusion: Severe stroke patients with malnutrition experienced an increased risk of death compared to those without malnutrition. GNRI, as a simple and practical nutritional screening tool, can be used as a routine approach to the nutritional status of stroke patients.

## 1. Introduction

Stroke has been the leading cause of death in China [1] and the second-leading cause of death globally [2]. In the United States, mortality from stroke was the fourth leading cause of death, and stroke was a leading cause of long-term severe disability [3]. During the past three decades, the absolute number of incidents and prevalent strokes increased to above 70% [2]. Despite the use of endovascular therapy and intravenous t-PA, the proportion of poor outcomes in patients with severe stroke remained high [4,5]. In patients with moderate-to-severe acute ischemic stroke (National Institutes of Health Stroke Scale (NIHSS) score above 8), the mortality at 90 days is about one in five. The proportion of severe disability or death (a modified Rankin score > 3) in patients with critical ill stroke (NIHSS > 20) is as high as 60% at 90 days [6]. Therefore, it is imperative to identify risk factors, especially modifiable clinical characteristics, to perform interventions to reduce the risk of poor outcomes in patients with severe stroke.

Although malnutrition is common in the elderly and is a poor prognostic risk factor for many diseases [7], it is still easily unrecognized and underestimated by clinicians. Previous studies have found that malnutrition is associated with increased long-term mortality in patients with cardio-cerebrovascular diseases, including acute coronary syndrome [8], heart failure [9], and acute ischemic stroke [10]. The prevalence of malnutrition after acute stroke varies widely, ranging from 6.1% to 62% depending on the patients’ characteristics, different timing, and method of assessment [11]. Even in high-income countries, the prevalence of malnutrition at admission in stroke patients is about 9% to 20% [12]. A recent study indicated that among Chinese patients with acute ischemic stroke, the prevalence of malnutrition ranges from 30.6% to 60.5% depending on the screening method used [13]. Furthermore, patients with severe stroke, especially those requiring ICU admission, were often at higher risk of malnutrition due to factors such as decreased level of consciousness and severe swallowing disorders [14]. Undernourished patients were more likely to develop pneumonia, other infections, and gastrointestinal bleeding during their hospital admission than other patients. A recent study has further found that hypocaloric nutrition in the early phase of a severe stroke may be associated with increased mortality [15]. Given that malnutrition is a modifiable risk factor, it is necessary to promptly assess the nutritional status of critically ill stroke patients through a convenient and precise method.

Therefore, this study aimed to assess the nutritional status of critically ill stroke patients at the time of ICU admission using the geriatric nutritional risk index (GNRI) and to clarify the effect of malnutrition defined by GNRI on outcomes in the early phases of severe stroke patients.

## 2. Materials and Methods

### 2.1. Study Population

All data are from the Medical Information Mart for Intensive Care (MIMIC)-III database [16], which was developed by the Laboratory for Computational Physiology at MIT. MIMIC-III contains clinical data from 58,976 ICU hospitalization records for 46,520 patients at Beth Israel Deaconess Medical Center from 2001 to 2012. The electronic health record (EHR) is maintained as a relational database that includes patient demographics, laboratory tests, bedside monitoring, diagnostic information (documented by International Classification of Diseases, Ninth Revision (ICD-9) codes), and in-hospital and out-of-hospital mortality. The use of the MIMIC-III database was approved by the review boards of the Massachusetts Institute of Technology and Beth Israel Deaconess Medical Center (Record ID 45811370).

Adult patients with a diagnosis of stroke (ICD-9 code between 430.00 and 434.91) for their first ICU admission record were included in this study. Both hemorrhagic and ischemic strokes are included. Since patients with severe stroke may have both ischemic and hemorrhagic stroke ICD-9 codes (e.g., cerebral infarction with hemorrhagic transformation, or cerebral infarction secondary to subarachnoid hemorrhage), if this is the case, we take the one with the superior diagnostic sequence as the primary diagnosis. We excluded patients only with lacunar cerebral infarction or post-stroke sequelae by filtering for ICD-9 codes. To identify the geriatric nutritional risk index (GNRI) scores, we also excluded patients without enough data (weight, height, and serum albumin level).

### 2.2. Malnutrition Screening Tools and Endpoints Assessment

The geriatric nutritional risk index (GNRI) was used to assess the nutritional status of stroke patients at their admission to the ICU. GNRI was calculated as follows: GNRI = 1.489 × serum albumin level (g/L) + 41.7 × (actual body weight (kg)/ideal body weight (kg)) [14]. The ideal body weight was defined as [height (m)]^2^ × 22. Concerning previous studies in the literature, we defined patients with high GNRI scores (>98) were at no risk of malnutrition, while those with a low GNRI (≤98) are at risk of malnutrition [17,18].

The primary endpoint of the study was mortality within 28 days of patient admission to the ICU, and the secondary endpoints were mortality within 90 days and 1 year after admission to the ICU, and the length of stay in the ICU. All patient follow-up information was obtained through the MIMIC-III database.

### 2.3. Data Extraction

We used Structured Query Language (SQL) statements to extract admission information. The following parameters were selected for further analysis: (1) basic demographic indicators: age, sex, height, weight, admission time; (2) disease severity score: sequential organ failure assessment (SOFA) score, simplified acute physiology score (SAPS); (3) comorbidities: coronary heart disease, atrial fibrillation, COPD, renal disease, liver disease, heart failure, malignancy, and sepsis; (4) vital Signs: mean arterial pressure, body temperature, and heart rate; (5) laboratory parameters: hemoglobin, leukocytes, platelets, creatinine, potassium, sodium, chloride, creatinine, lactate, pH, and serum albumin; (6) treatment: thrombolysis, endovascular therapy, and sedative use.

### 2.4. Statistical Methods

Propensity score matching (PSM) was used to balance the differences in baseline characteristics between patients who were at risk of malnutrition (GNRI ≤ 98) and those who did not (GNRI > 98). A propensity score was estimated using multivariable logistic regression based on various factors at admission, which were age (continuous), sex (male vs. female), disease severity score (simplified acute physiology score (SAPS); continuous), comorbidities (atrial fibrillation, diabetes, COPD, renal disease, liver disease, malignancy, and sepsis; each comorbidity entered in the model as dummy variable), and vital signs (mean arterial pressure, body temperature, and heart rate; continuous). One-to-one nearest-neighbor caliper matching was used to match patients based on the logit of the propensity score using a caliper equal to 0.01. To evaluate the effectiveness of the propensity score model in balancing the two compared groups, the imbalance of covariates for the original (non-matched) and the adjusted (matched) cohorts was compared.

Continuous data were reported as mean and standard deviation (SD) or median (interquartile range) based on whether the normal distribution is met. Categorical variables were expressed as numbers (%). The Student’s *t*-test or Wilcoxon signed-rank test was used to statistically test the differences among the continuous covariates where appropriate. A chi-square test was used to test the differences among the categorical covariates.

The Kaplan–Meier method was used to analyze the survival data, and the log-rank test was used for comparison. We used a Cox proportional hazards regression to elucidate association between GNRI groups and mortality at 28 days, 90 days, and 1 year for patients in the matched cohorts. Further, we constructed the restricted cubic spline with 5 knots to flexibly represent the association between the risk of 28-day/90-day/1-year mortality and GNRI as a continuous variable.

### 2.5. Sensitivity Analyses

We performed two sensitivity analyses to assess the robustness of our main estimates. Firstly, in the original cohort, we used multivariable Cox hazard regression models to investigate the association between GNRI groups and outcomes. Model 1 was adjusted for age, and gender. Model 2 was adjusted for model 1 plus SAPS score, SOFA score, vital signs (heart rate, temperature, and MAP), and complications (atrial fibrillation, COPD, CHD, diabetes, sepsis, liver disease, and malignancy). Model 3 adjusted for model 2 plus Hb, WBC, platelet, sodium, potassium, BUN, creatinine, chloride, and bicarbonate. Secondly, to further assess the impact of potential confounding after PSM, we adjusted for residual baseline imbalances that remained despite matching with a conditional Cox proportional hazards model.

Two-sided *p* < 0.05 was considered statistically significant for all tests. All analyses were conducted using the R tool (version 3.6.3, R Foundation for Statistical Computing, Vienna, Austria), Stata software (version 16, Stata Corporation LLC, College Station, TX, USA), and SPSS software (version 23.0, IBM, NY, USA).

## 3. Results

### 3.1. Patient Characteristics

**Figure 1:**
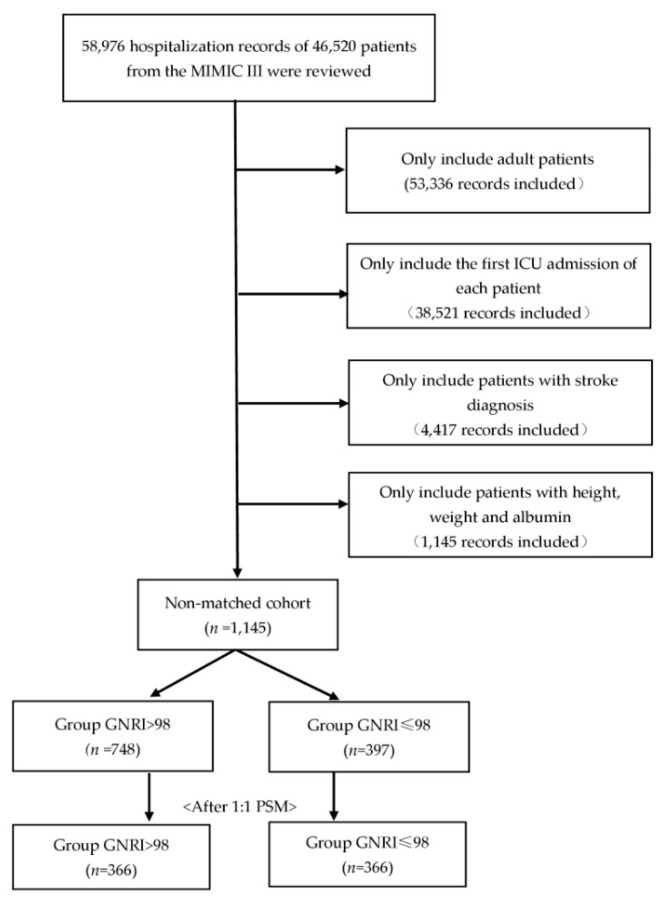
The patients’ selection process in the MIMIC III database.

After reviewing 58,976 hospitalization records from MIMIC-III, we identified 4417 patients with critically ill stroke, and 3272 records were excluded due to the absence of height, weight, or albumin. Of the remaining 1145 patients, a total of 748 (65%) individuals were in the high GNRI group, representing no risk of malnutrition, and 397 (35%) were in the at-risk group (GNRI ≤ 98). After 1:1 propensity score matching, a total of 732 patients were dichotomized into low GNRI or high GNRI groups. Detailed information on patient selection is presented in Figure 1.

The admission profiles of the two groups are described in Table 1. Overall, the mean age was 66.7 years (SD 15.3) and 56% of the patients were male. The most common comorbidities among them were atrial fibrillation (32.1%), coronary heart disease (30.6%), diabetes (28.7%), and congestive heart failure (24.9%). Among non-matched cohorts, there were 641 (56%) ischemic stroke and 504 (44%) hemorrhagic stroke patients. Patients in the low GNRI group (≤98) were older, thinner, had higher scores for disease severity, and were more likely to suffer from anemia, chronic heart failure, COPD, or sepsis (all *p* < 0.05). As shown in Table 1, most of the covariates of the matched cohorts were similar between the two groups, except for weight, body mass index (BMI), serum albumin, hemoglobin, and two comorbid conditions (coronary heart disease and AIDS/HIV).

### 3.2. Association between Different GNRI Groups and Mortality

In the nonmatched cohort, the low GNRI group suffered higher mortality compared with the high GNRI group at 28 days (28.7 vs. 16.7%), 90 days (36.5 vs. 21.3%), and 1 year (44.3 vs. 26.2%) (*p* < 0.001). Still, the propensity score-matched mortality rates for the low GNRI groups were significantly higher than the high GNRI group in all observation time points, respectively, at 28 days (27.9 vs. 20.8%), 90 days (35.5 vs. 25.7%), and 1 year (43.4 vs. 30.9%) (all *p* < 0.05) (Table 1).

**Figure 2:**
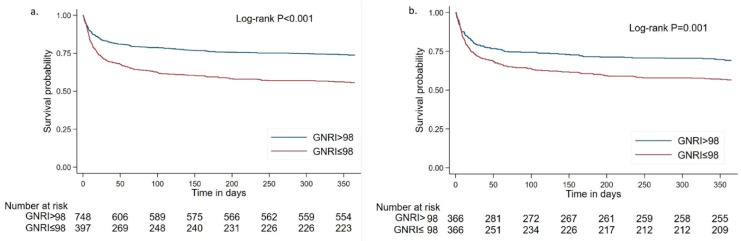
Kaplan–Meier curves for severe stroke patients with different GNRI groups. Survival probability for 1 year in the total (nonmatched) cohort (**a**) and the matched cohort (**b**).

The survival curve with 1-year follow-up for patients with different GNRI groups is shown in Kaplan–Meier plots in Figure 2. The Kaplan–Meier analysis found that survival probability was lower in the low GNRI group compared with the high GNRI group (log-rank test *p* < 0.001). To better visualize the survivorship of different GNRI groups during the acute-phase of onset, we also plotted Kaplan–Meier curves at different follow-up time-points (see Supplementary materials; Figure S1). We found that the statistical difference is not only in an acute stage but also in long-term outcomes. This finding is still robust in the matched cohort (log-rank test *p* < 0.01; Figure 2).

We used Cox regression models to determine the association between the different GNRI groups and mortality among patients with severe stroke (Table 2). The high GNRI group (>98) was always considered the reference group. Compared with those in the high GNRI group, patients in the low GNRI group were associated with a 1.38-fold increase in the hazard of 28-day mortality (95% CI 1.03–1.86), 1.45-fold increase in 90-day mortality (95% CI 1.11–1.89), and 1.51-fold increase in 1-year mortality (95% CI 1.19–1.92; Table 2). During the follow-up, patients with low GNRI presented with a higher hazard of all-cause mortality over time.

**Figure 3:**
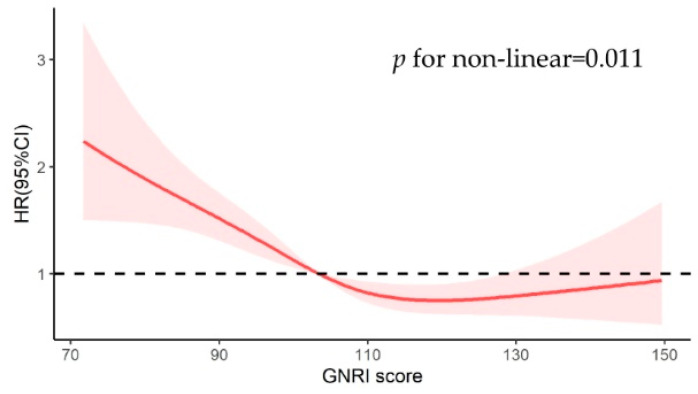
Relationship between GNRI score as a continuous variable and 1-year mortality in the matched cohort. A thick red line indicates hazard ratio estimates, with reddish shaded areas showing 95% confidence intervals derived from the restricted cubic spline. The reference line is indicated by a solid bold line at a hazard ratio of 1.0.

To explore the relation between GNRI as a continuous variable and mortality in patients with severe stroke at different time points, we constructed restricted cubic splines with GNRI 98 as a reference (Figure 3). As shown in the figure, the relationship between admission GNRI scores and all-cause mortality was non-linear (*p* = 0.036). The GNRI scores at which the hazard ratio for mortality plateaus was approximately 110–130. When the GNRI score is below 110, the risk of death increases rapidly with the decrease in GNRI. 0

### 3.3. Sensitivity Analyses

To control the potential impact of residual and/or unmeasured confounding of propensity score-matched analysis, we performed doubly robust analysis of association between different GNRI groups and mortality. These analyses were consistent with the primary results (see Supplementary materials; Tables S1 and S2). Multivariable Cox hazard regression models adjusting for various potential confounders of the original (non-matched) cohorts were performed, and the findings were similar to our main reported estimates. For example, the hazard ratio for the association between low GNRI and 28-day mortality was 1.45 (95% CI 1.09–1.92; Table S1).

## 4. Discussion

In this retrospective cohort study, we assessed the relationship between nutritional status at admission and clinical outcomes at 28 days, 3 months, and 1 year of onset in patients with severe stroke. We found severe stroke patients who were undernourished suffered increased mortality compared to those who were not at risk of malnutrition, and this finding still holds after adjusting for multiple variables.

Malnutrition is common, especially in the senior population. Wei et al. report that malnutrition accounts for 12.6% of the general elderly population in China [19]. The prevalence of malnutrition among stroke patients ranged from 8% to 60.5% depending on the various approaches to malnutrition [13,20]. Early recognition of malnutrition significantly affects the outcome [21]. In contrast, many clinicians have overlooked the increased risk of mortality associated with malnutrition or have found it difficult to implement because of the complexity of screening approaches.

An association between malnutrition, as defined by other approaches, and poor prognosis of stroke has also been found in previous studies [10,13], while patients at risk of malnutrition in these studies had a more advanced age and more comorbidity. These characteristics might be confounding factors in increased mortality. Given this, we used a propensity score matching method to reduce the bias in estimating the effect of malnutrition on post-stroke outcomes, and the likelihood of confounding when analyzing the observational data [22]. According to previous studies, there was a relatively significant imbalance in the original cohort between the two groups. Undernourished patients are older and had poorer laboratory features and disease severity. After matching, malnutrition defined by GNRI still harmed outcomes including increased mortality and length of ICU stay.

GNRI is an integration of serum albumin and body mass index (BMI); therefore, this constitutive relation may explain the association between malnutrition in stroke patients and increased mortality. Serum albumin is a multifunctional protein that plays important neuroprotective roles in stroke [23]. It constitutes a major antioxidant defense against oxidizing agents and reverses stagnation within cortical venules in the reperfusion phase after focal ischemia [24,25]. Low serum albumin is a signal of increased venous thromboembolism, which indicates a state of high inflammation or hyper-coagulation [26]. Therefore, low serum protein has been found in previous studies to be significantly associated with poor prognosis in stroke [27], especially in the cardioembolic stroke subtype [28]. As well, several studies have discussed the relationship between BMI and mortality or prognosis after stroke onset. Obesity is an established risk factor for stroke, but previous studies also found the so-called “obesity paradox”. That is, patients with higher BMI have a survival advantage over those with low BMI after the onset of stroke [29]. Potential mechanisms of poor prognosis of low BMI patients include inadequate nutritional reserves during the recovery period and a higher frequency of thromboembolic infarction [30].

A variety of nutritional assessment approaches are available, among which GNRI is a worthwhile one for its convenience. Nutritional Risk Screening 2002(NRS2002) and Nutrition Risk in the Critically Ill (NUTRIC) Score are commonly used in ICU patients. NRS2002 [31] requires subjective recall of weight change in the last three months; however, it is difficult or inaccurate for stroke patients with cognitive impairment, visual and hearing impairment, or aphasia. NURTIC [32] requires the APACHII score (with 18 variables for calculation), which may be unfamiliar to neurologists and potentially difficult to implement because of its complexity. Compared to these, GNRI can be calculated by measuring only height, weight and a simple blood sample, making it easier and faster for clinicians to use.

Timely and appropriate nutrition support has been shown to improve outcomes and decrease mortality [33]. In an RCT of nutrition protocols for severe stroke patients requiring ICU admission, it was found that older patients, who had lower BMI and were at higher nutritional risk may benefit more from full energy therapy [12]. In contrast to early nutrition strategies in patients with sepsis or ARDS, a more aggressive early feeding strategy compared with low-calorie enteral nutrition may result in the survival of patients with severe stroke. Therefore, early identification of patients at risk for malnutrition and aggressive interventions is significant since they will benefit from targeted nutritional supplementation.

Our study has several limitations that should be considered. Firstly, as a single-center study based on electronic record data, there may be potential confounding of variables not recorded in the electronic health record and the generalizability of the findings needs further validation in other institutions. Secondly, since this study was retrospective, selection bias is inevitable in study designs. However, a sensitivity analysis was carried out to support the consistency of our results. Future prospective studies will help to validate our findings. In addition, due to the limitation of the record types of the MIMIC database, our outcomes did not include additional relevant endpoints such as the recurrence of stroke or the Modified Rankin Scale for neurologic disability. There was only all-cause mortality, without specific causes of death. Future research should seek to further depict the full stroke outcome spectrum caused by malnutrition.

## 5. Conclusions

In conclusion, we found that malnutrition is highly prevalent in patients with severe stroke, and the low GNRI was associated with an increased risk of all-cause mortality. GNRI, as a simple and practical nutritional screening tool, can be used as a routine approach to the nutritional status of stroke patients, facilitating the classification of patients who need more aggressive nutritional support.

## Figures and Tables

**Table 1:** Characteristics and outcomes of participants categorized by GNRI.

	Non-Matched Cohort			Matched Cohort		
Characteristics	High GNRI Group (≥98)	Low GNRI Group (<98)	*p*	High GNRI Group (≥98)	Low GNRI Group (<98)	*p*
*n*	748	397	NA	366	366	NA
Age, years	65.17 ± 15.12	69.51 ± 15.29	**<0.001**	69.63 ± 13.18	69.12 ± 15.42	0.49
Male	427 (57.1%)	215 (54.2%)	0.34	201 (54.9%)	198 (54.1%)	0.82
Weight, kg	87.55 ± 21.05	66.56 ± 13.59	**<0.001**	86.03 ± 21.40	66.80 ± 13.66	**<** **0.001**
BMI	30.34 ± 6.45	23.27 ± 3.57	**<0.001**	31.11 ± 6.45	23.32 ± 3.59	**<** **0.001**
SAPS score	18.69 ± 4.9	20.65 ± 5.06	**<0.001**	20.27 ± 4.68	20.23 ± 4.81	0.13
SOFA score	4 (2–6)	5 (3–7)	**<0.001**	4 (2–7)	4 (3–7)	0.38
GNRI score	112.57 ± 11.77	88.39 ± 7.42	**<0.001**	111.46 ± 11.51	88.49 ± 7.31	**<** **0.001**
**Types of stroke**						
Ischemic stroke	413 (64%)	228 (36%)	NA	207 (48%)	221 (52%)	NA
Hemorrhagic stroke	335 (66%)	169 (34%)	NA	159 (52%)	145 (48%)	NA
**Comorbidities**						
CHF	170 (22.7%)	115 (29.0%)	**0.02**	94 (25.7%)	106 (29.0%)	0.32
Renal	68 (9.1%)	51 (12.9%)	0.05	36 (9.8%)	44 (12.0%)	0.34
AFIB	241 (33.2%)	127 (32.0%)	0.94	123 (33.6%)	118 (32.2%)	0.69
Liver	18 (2.4%)	15 (3.8%)	0.19	12 (3.3%)	11 (3.0%)	0.83
COPD	57 (7.6%)	55 (13.9%)	**0.001**	46 (12.6%)	40 (10.9%)	0.49
CHD	247 (33.0%)	103 (25.9%)	**0.01**	118 (32.2%)	92 (25.1%)	**0.03**
Malignancy	100 (13.4%)	66 (16.6%)	0.14	52 (14.2%)	52 (14.2)	1.00
AIDS	0 (0%)	5 (1.3%)	**0.005**	0 (0%)	4 (1.1%)	**0.045**
Diabetes	237 (31.68%)	92 (23.2%)	**0.002**	93 (25.4%)	89 (24.3%)	0.73
Sepsis	51 (6.8%)	52 (13.1%)	**<0.001**	37 (10.1%)	40 (10.9%)	0.72
**Vital signs**						
Heart rate	82.64 ± 17.77	86.47 ± 19.82	**<0.001**	84.83 ± 19.40	85.01 ± 18.99	0.75
MAP	87.12 ± 19.64	86.54 ± 19.68	0.63	87.54 ± 21.42	86.89 ± 19.44	0.33
Temperature (°C)	36.6 (36.0–37.1)	36.6 (35.9–37.1)	0.42	36.6 (35.9–37.0)	36.6 (35.9–37.1)	0.69
**Lab tests**						
Serum albumin, g/dL	3.7 (3.4–4.0)	3.0 (2.6–3.4)	**<0.001**	3.7 (3.3–4.0)	3.0 (2.6–3.4)	**<0.001**
WBC	11.35 (8.50–14.50)	11.30 (8.20–15.40)	0.86	11.80 (9.00–15.05)	11.05 (8.10–15.20)	0.07
Hb	11.70 (9.80–13.10)	10.50 (9.30–12.08)	**<0.001**	11.45 (9.28–12.90)	10.60 (9.40–12.03)	**0.003**
Platelet	200.00 (149.00–262.00)	206.00 (147.00–268.00)	0.70	197.00 (151.75–260.00)	203.50 (146.25–269.00)	0.56
Sodium	138.00 (136.00–141.00)	139.00 (135.00–142.00)	0.46	138.00 (136.00–141.00)	139 (135.00–142.00)	0.51
Potassium	4.00 (3.60–4.40)	3.90 (3.60–4.40)	0.17	4.00 (3.60–4.50)	3.90 (3.60–4.40)	0.08
Bicarbonate	24.00 (22.00–26.00)	23.00 (21.00–26.00)	**0.008**	23.00 (21.00–26.00)	23.00 (21.00–25.00)	0.18
Chloride	106.00 (102.00–108.00)	106.00 (102.00–110.00)	**0.015**	106.00 (102.00–109.00)	107.00 (102.00–110.00)	0.07
BUN	16.00 (12.00–24.00)	18.00 (13.00–29.50)	**<0.001**	18.00 (13.00–27.00)	18.00 (13.00–28.00)	0.93
Creatinine	0.90 (0.70–1.20)	0.90 (0.70–1.40)	0.258	0.90 (0.70–1.30)	0.90 (0.70–1.40)	0.41
**Interventions**						
Sedative use	452 (60.4%)	241 (60.7%)	0.93	243 (66.4%)	222 (60.7%)	0.11
Infusion of thrombolytic agent	49 (6.6%)	19 (4.8%)	0.23	20 (5.5%)	18 (4.9%)	0.21
Endovascular removal of obstruction	18 (2.4%)	14 (3.5%)	0.27	11 (3.0%)	14 (3.8%)	0.20
**Clinical Outcomes**						
Mortality_ 28-day	125 (16.7%)	114 (28.7%)	**<0.001**	76 (20.8%)	102 (27.9%)	**0.025**
Mortality_ 90-day	159 (21.3%)	145 (36.5%)	**<0.001**	94 (25.7%)	130 (35.5%)	**0.004**
Mortality_ 1-year	196 (26.2%)	176 (44.3%)	**<0.001**	113 (30.9%)	159 (43.4%)	**<0.001**
ICU LOS(d)	8.23 ± 8.72	9.77 ± 9.70	**0.006**	8.32 ± 0.46	9.86 ± 0.51	**0.024**

BMI, body mass index; SAPS, simplified acute physiology score; SOFA, sequential organ failure assessment; CHF, congestive heart failure; AFIB, atrial fibrillation; CHD, coronary heart disease; COPD, chronic obstructive pulmonary disease; MAP, mean arterial pressure; Hb, hemoglobin; WBC, white blood cell count; BUN, blood urea nitrogen. ICU LOS, ICU length of stay. Bold values indicate statistical significance (*p* < 0.05).

**Table 2:** Association between GNRI groups and the mortality of severe stroke patients in propensity-score matched cohort.

Outcomes	Hazard Ratio (95% CI)	*p* Value
28-day mortality	1.38 (1.03–1.86)	0.03
90-day mortality	1.45 (1.11–1.89)	0.006
1-year mortality	1.51 (1.19–1.92)	0.001

Hazard ratio and 95% CI for the GNRI group in mortality at 28 days, 90 days, and 1 year mortality were calculated based on a Cox proportional hazard model. The high GNRI group (>98) was always considered the reference group. CI, confidence interval.

## Data Availability

Publicly available datasets were analyzed in this study. The data can be found here: https://physionet.org/content/mimiciii/1.4/ (accessed on 5 October 2022).

## Supplementary Materials

The following supporting information can be downloaded at: https://www.mdpi.com/article/10.3390/nu14224786/s1, Figure S1. Kaplan-Meier curves for severe stroke patients with different GNRI groups at short-term of onset. Table S1. Association between GNRI group and the outcomes of severe stroke patients (in non-matched cohort). Table S2. Hazard ratio and 95% CI for the GNRI group in mortality, adjusting for variables that remained unbalanced between the groups in the propensity score model.
