# Supplementary material for: Malnutrition Defined by Geriatric Nutritional Risk Index Predicts Outcomes in Severe Stroke Patients: A Propensity Score-Matched Analysis
Source: Nutrients. 2022 Nov 12;14(22):4786. doi: 10.3390/nu14224786 (PMC9696179; doi:10.3390/nu14224786)
Supplement: Supplementary file 1 [file nutrients-14-04786-s001.zip › nutrients-1981711-supplementary.pdf]

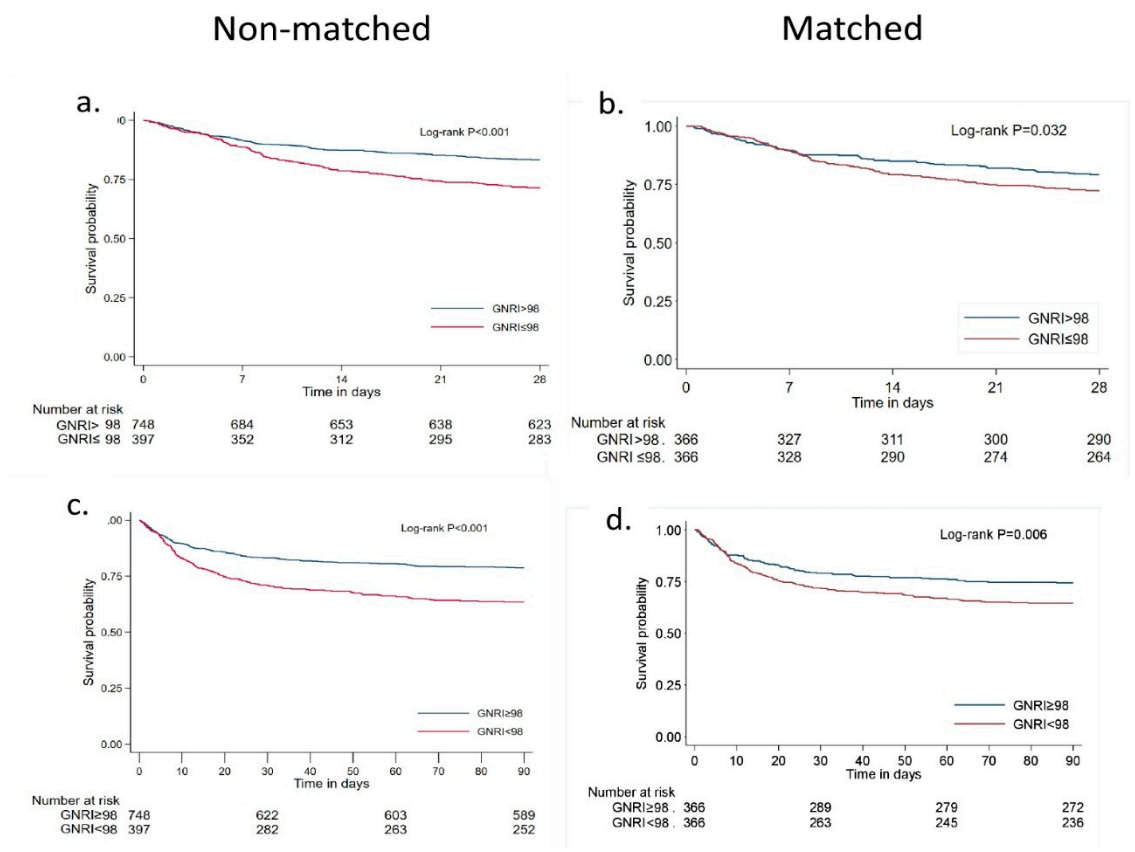

Figure S1. Kaplan-Meier curves for severe stroke patients with different GNRI groups at short-term of onset. Survival probability in the total (nonmatched) cohort for 28-day (a.) and 90-day(c.). Survival probability in the matched cohort for 28-day (b.) and 90-day(d.)

Table S1. Association between GNRI group and the outcomes of severe stroke patients (in non-matched cohort)

Model	28-day mortality		90-day mortality		1-year mortality	
	Hazard ratio (95% CI)	*p* value	Hazard ratio (95% CI)	*p* value	Hazard ratio (95% CI)	*p* value
Model 1	1.716(1.328-2.218)	＜0.001	1.756(1.399-2.204)	＜0.001	1.753(1.427-2.153)	＜0.001
Model 2	1.404(1.076-1.831)	0.001	1.459(1.152-1.848)	0.002	1.449(1.169-1.794)	0.001
Model 3	1.445(1.090-1.915)	0.01	1.532(1.194-1.965)	0.001	1.523(1.224-1.895)	＜0.001

Hazard ratio and 95% CI for the GNRI groups in mortality at 28 days, 90 days, and 1-year mortality were calculated using different Cox regression models. The high GNRI group (>98) was always considered the reference group.

Model 1 adjusted for age, and gender. Model 2 adjusted for model 1 plus SAPS score, SOFA score, vital signs (heart rate, temperature, and MAP), and complications (atrial fibrillation, COPD, diabetes, sepsis, liver disease, and malignancy). Model 3 adjusted for model 2 plus Hb, WBC, platelet, sodium, potassium, BUN, creatinine, chloride, and bicarbonate.

CI, confidence interval; SAPS, simplified acute physiology score; SOFA, Sequential Organ Failure Asses; MAP, mean arterial pressure; COPD, chronic obstructive pulmonary disease; Hb, Hemoglobin; WBC, white blood cell count; BUN, blood urea nitrogen.

Table S2. Hazard ratio and 95% CI for the GNRI group in mortality, adjusting for variables that remained unbalanced between the groups in the propensity score model

	28-day mortality		90-day mortality		1-year mortality	
	Hazard ratio (95% CI)	*p* value	Hazard ratio (95% CI)	*p* value	Hazard ratio (95% CI)	*p* value
**Overall**	1.42(1.04-1.93)	0.03	1.44(1.10-1.89)	0.008	1.50(1.18-1.92)	0.001

Based-on conditional Cox proportional hazards model, adjusting for variables that remained unbalanced between the groups in the propensity score model
